# Multi-Model Ensemble Projections of Winter Extreme Temperature Events on the Chinese Mainland

**DOI:** 10.3390/ijerph19105902

**Published:** 2022-05-12

**Authors:** Xiuping Yi, Ling Zou, Zigeng Niu, Daoyang Jiang, Qian Cao

**Affiliations:** 1 Hunan Key Laboratory of Remote Sensing of Ecological Environment in Dongting Lake Area, School of Geography and Information Engineering, China University of Geosciences, Wuhan 430074, China; yixiuping@cug.edu.cn (X.Y.); nzg@cug.edu.cn (Z.N.); jiangdaoyang1010@163.com (D.J.); caoqian@cug.edu.cn (Q.C.); 2 Key Laboratory of Regional Ecology and Environmental Change, State Key Laboratory of Biogeology and Environmental Geology, China University of Geosciences, Wuhan 430074, China; 3 Jülich Supercomputing Centre, Forschungszentrum Jülich, 52428 Jülich, Germany

**Keywords:** climate change, downscaling, extreme temperatures, population exposure, warm winters

## Abstract

Based on the downscaling data of multi-model ensembles of 26 global climate models (GCMs) from the Coupled Model Intercomparison Project Phase 6, this study calculated the extreme climate indices defined by the Expert Team on Climate Change Detection and Indices and the warm winter extreme grade indices to explore winter climate response on the Chinese mainland under different shared socioeconomic pathways (SSPs) and representative concentration pathways. The results showed that the temperature in winter increased overall, with the highest temperature increases of 0.31 °C/10a (Celsius per decade) (SSP245) and 0.51 °C/10a (SSP585) and the lowest temperature increases of 0.30 °C/10a (SSP245) and 0.49 °C/10a (SSP585). Warm-related extreme weather events such as warm days and warm spell duration indices showed an increasing trend, whereas cold-related extreme weather events such as cold spell duration indices, cold nights, ice days, and frost days showed a decreasing trend. On the regional scale, the maximum temperature increased by more than 2 °C/10a (SSP245) and 0.4 °C/10a (SSP585), except in South China, and the minimum temperature increased faster in Qinghai-Tibet and Northeast China compared to elsewhere on the Chinese mainland. Compared with that under SSP585, the frequency and intensity of warm winters in the latter half of the 21st century were lower under SSP245. At the end of the 21st century, under the SSP245 scenario, warm winter frequency in most regions will be reduced to below 60%, but under the SSP585 scenario, it will be more than 80%. Population exposures all showed a downward trend, mainly due to the reduction of warm winter events and the decline of the population under the SSP245 and SSP585 scenarios, respectively. If the greenhouse gas emission path is controlled in the SSP245 scenario, the population exposure risk in warm winters can be decreased by 25.87%. This study observed a consistent warming trend on the Chinese mainland under all SSPs in the 21st century and found that stricter emission reduction policies can effectively decrease the population exposure to warm winters.

## 1. Introduction

Over the past 100 years since the industrial revolution, climate change has become a growing concern and has had a powerful impact on human daily life [1,2]. Agricultural production, personal health, and environmental protection are affected by global warming, which can be attributed to extreme temperature and precipitation events caused by climate change. In the context of the global warming scenario, increasing temperature trends are observed in each season, especially in winter [3,4,5,6].

Indices defined by the Expert Team on Climate Change Detection and Indices (ETCCDI) have been utilised to evaluate regional- and national-scale temperature and precipitation extremes in many studies [7]. The Coupled Model Intercomparison Project Phase 5 (CMIP5) projected future extreme weather events for the Chinese subregion based on six ETCCDI, and the results showed that cold nights (TN10P) and frost days (FD0) decreased significantly in the 21st century, with a faster decline in the late 21st century than the middle of the 21st century. In contrast, warm days (TX90p), tropical days (TD30), and daily maximum temperature (TXx) increased under both representative concentration pathway (RCP) 4.5 and RCP 8.5 scenarios [8]. Changes in winter extreme low-temperature events in northern China under different future warming scenarios were predicted based on a simple minimum complexity earth simulator model [9]. This showed that the intensity of low-temperature events will decrease in the future because of higher velocity of TNn, especially in Northeast China (NE), where TNx increased by 0.1 °C/0.6 °C compared to 1.5 °C/2.0 °C for TXx. The temperature prediction results based on CMIP5 by You et al. showed that under the RCP8.5 scenario, the mean temperature, maximum and minimum temperature had highest warming rate compared with the RCP4.5 and RCP2.6 scenarios. Northeast China and the Qinghai-Tibet Plateau are more vulnerable to climate change in future emission scenarios [10]. A statistical model of extreme climates events based on generalised extreme value distribution was applied to CMIP5 predictions. The extreme high temperature in China was expected to increase by about 1.66–4.92 °C. The variation of the warm extreme value was more sensitive to the radiative forcing concentration than the cold extreme value [11]. Chen et al. proposed that the credibility of the model can be improved by using a multi-model ensemble. The overall performance is better than a single model when the mean and median of the multiple models are considered. Model assessments vary widely across indices based on relative errors in climatology [12]. Validation with observations and reanalysis showed that compared with CMIP5, the CMIP6 model had obvious advantages in terms of spatial patterns and temporal changes in extreme temperature indices [1]. Previous studies have shown that winter temperature is a vital limiting factor for agricultural production and vegetation growth [13]. China is one of the world’s largest agricultural countries. Chen et al. [14] investigated the effects of climate change and extreme climate on maize and rice growth based on climate variable outputs from 17 general environment models (GCMs) in the CMIP5 dataset in the Yangtze River Basin. Their results showed that the extreme climate index was closely correlated with maize and rice yields, particularly on days above the temperature threshold. Thus, studying the spatiotemporal variation of climate in winter is beneficial for agricultural pattern modification and phenological adaptation. However, little research has been conducted on extreme winter temperatures and the overall warm bias.

Future climate can be predicted using global climate models made up of equations describing the interaction of energy and matter between different parts of the ocean, atmosphere, and land [15,16]. The Coupled Model Intercomparison Project Phase 6 (CMIP6) announced in 2016 is the latest GCM dataset, which is reliable for projecting the future climate. Compared with previous versions, CMIP6 has a higher resolution and a higher correlation with high-resolution daily observations of the Chinese mainland from 1961 to 2005 [17]. However, the resolution of CMIP6 at a scale of hundreds of kilometres is too coarse for studying extreme climate events and climate change in small regions [18,19]. There are three commonly used downscaling methods for converting crudely generated data to site scale depending on research needs: scaling, dynamical downscaling, and statistical downscaling. Compared to the statistical downscaling method, the scaling method cannot explain multiple climate variabilities, whereas the dynamic downscaling method cannot efficiently and rapidly calculate large areas [20,21,22]. Because of its high accuracy and low cost, the statistical downscaling method is often utilised to predict future climate with high accuracy and resolution. NWAI-WG, developed by Liu and Zuo [23] is a widely applied statistical downscaling method that uses daily observational climate data series to improve accuracy instead of circulation data. In terms of application, Wang et al. [24] analysed the extreme weather event indices of the wheat growing belt in southeast Australia under different RCPs through multi-model ensemble data derived from the NWAI-WG downscaled CMIP5 data. They discovered an upward trend for warm days, warm nights, and hot days and a downward trend for frost days and cold nights. Xiao et al. [25] calculated future temperature extremes in the region of the Han River basin using the NWAI-WG downscaled CMIP5 multi-model ensembles. The results showed that the variation range of the minimum temperature extreme value was greater than that of the maximum temperature extreme value, and that the mean value was asymmetrical to the variation in daytime and night-time extreme values. Tang and Liu [26] assessed the historical and future climate stability of summer maize using the NWAI-WG downscaled CMIP5 data from the period 1996–2100. Li et al. [27] analysed historical extreme precipitation events based on NWAI-WG statistical downscaling data and estimated extreme precipitation events in future scenarios.

Although research on site-scale extreme temperature events during winter on the Chinese mainland could be helpful in coping with winter climate change, few studies have been conducted on this. This study provides an initial overview of a warm winter on the Chinese mainland through multi-model ensembles under SSP245 and SSP585 scenarios that represent moderate greenhouse gas emissions with strict regulation and high greenhouse gas emissions with low binding management, respectively. Monthly CMIP6 grid data obtained from WRCP were downscaled to daily site-scale data using the NWAI-WG method combined with site observation data. Future winter extreme weather events and overall seasonal warm/cold biases were predicted using the methods announced by the ETCCDI and NCC based on the multi-model ensemble mean from CMIP6. In this study, the temporal and spatial trends in extreme weather event frequency and intensity under different SSP scenarios were calculated.

## 2. Materials and Methods

### 2.1. Study Area and Data Sources

Observation station daily-scale data were provided by the Chinese National Meteorology Center. These data included maximum temperature, minimum temperature, and average temperature for the period 1961–2015. The CMIP6 data were collected from the CMIP6 website, which contain monthly data covering the period 1961–2100. The NWAI-WG statistical downscaling method developed by Liu and Zuo [23] was utilised to downscale the gridded CMIP6 data to the station scale, which can generate daily time-series data from monthly GCMs. The population grid point data (2010–2100) of the Chinese mainland under the Shared Socioeconomic Pathways (SSPs) used in this paper are from the team of Professor Jiang Tong, Institute of Disaster Risk Management, Nanjing University of Information Science and Technology.

**Figure 1:**
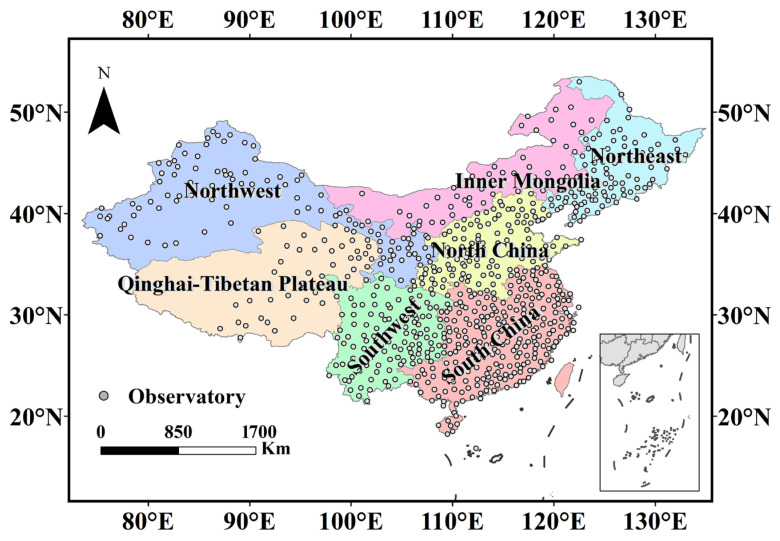
Study area.

The entire Chinese mainland (ECM) contains various topographical and climatic zones (Figure 1). The entire Chinese Mainland was divided into seven subregions according to climatic characteristics and administrative divisions. The seven subregions are Inner Mongolia (IM), North China (NC), Northeast China (NE), Northwest China (NW), Qinghai-Tibet plateau (QT), South China (SC), and Southwest China (SW).

### 2.2. Data Uncertainty Estimation

The uncertainty in greenhouse gas emission levels is an important source of uncertainty in future climate predictions. This is related to the uncertainty of future technological development and socioeconomic policies. Because the natural variability of climate caused by the uncertainty of climate change predictions primarily comes from solar radiation, solar, and tidal movement between parts of the climate regions at different time and space scales, there exists a complicated nonlinear interaction relation, resulting in great complexity or difficulty in predicting the climate and climate change. There are differences in simulation performance among different models because of the structural framework of the models, description of physics, and selection of physical parameters in the models [28]. The uncertainty in GCM predicted data is primarily due to three factors: model uncertainty (M(t)), scenario uncertainty (S(t)), and internal variabilities (V) [29]. The model predicted value $X_{m,s,t}$ is a combination of these three components: $x_{m,s,t}$ represents the smooth fit, $i_{m,s}$ represents the reference air temperature, and $\varepsilon_{m,s,t}$ represents the residual. Variation in these three components as the dominant factors of uncertainty is a function of time. The uncertainty estimation method used to quantify the uncertainty is as follows [30]:

(1) $$X_{m,s,t}=x_{m,s,t}+i_{m,s}+\varepsilon_{m,s,t}$$

(2) $$V=\sum \frac{1}{n}var_{s,t}\left(\varepsilon_{m,s,t}\right)$$

(3) $$M\left(t\right)=\frac{1}{N_{s}}\underset{s}{\sum }var_{m}\left(x_{m,s,t}\right)$$

(4) $$S\left(t\right)=var_{s}\left(\underset{m}{\sum }\frac{1}{n}x_{m,s,t}\right)$$

where *n* is the number of models (*m*) and Ns is the number of scenarios (*s*).

### 2.3. Extreme Temperature Indices (ETIs)

The monitoring, detection, and attribution of extreme climate change typically require daily resolution data. However, compilation, provision, and update of globally complete and readily available full-resolution daily datasets is a very difficult task. Therefore, the ETCCDI has been coordinating international developments to calculate and analyse a range of indicators to ensure that individuals, countries, and regions can calculate the indicators in exactly the same way [31,32,33]. We estimated the temperature indices announced by the ETCCDI (Table 1). These indices can be divided into two classes: relative and absolute. The relative indices are defined based on values higher or lower than the threshold designated from the percentile on the time-series data and include cold night (TN10P), hot day (TX90P), warm spell duration index (WSDI), and cold spell duration index (CSDI). The absolute indices are defined based on values higher or lower than a certain threshold and include FD0, ID0, minimum daily temperature (TNn), daily maximum temperature (TXx), and daily temperature range (DTR).

### 2.4. Warm Winter Index

A proper definition and uniform standard to classify warm winters is necessary for studying extreme climate change in winter seasons. Previously, warm winter was defined as average temperature anomaly in winter (December to February) exceeding 0.5 °C. However, many scholars have adopted different methods to determine warm winter, making the statistical analysis results to have large differences [34,35]. To analyse the warm bias in winter during the future time series, the standard warm winter proposed by the China Meteorological Administration (CMA), which was derived using a dynamic baseline method considering the warming scenario compared to the static-baseline method, was utilised in this study. Winter temperature is regarded as a Gaussian distribution in this method, and the threshold of winter warm bias is determined by the closest climatic condition, a 30-year moving average, and its standard deviation [36]. Climatological normal standard deviation and anomaly can be calculated using the following formulas:

(5) $$T_{a}=\frac{1}{30}\overset{30}{\underset{i=1}{\sum }}T_{i}$$

(6) $$\sigma =\sqrt{\frac{1}{29}\overset{30}{\underset{i=1}{\sum }}\left(T_{i}-T_{a}\right)}$$

(7) $$\Delta T=T-T_{a}$$

where *T_a_* represents the average climate temperature calculated from the temperature time series ($T_{i}$) for the last 30 years. The climatological normal and standard deviation as fiducials is defined as the results calculated from the closest three decades and updated every decade.

#### 2.4.1. Station Warm Winter

The trisection method was used to determine the threshold of warm winters for the meteorological stations. The winter average temperature is considered to follow a standard normal distribution for defining the threshold of warm and cold deviations, and the probability density distribution was divided from the third point. Hence, the thresholds for warm winter and strong warm winters are defined as anomalies more than 0.43σ and 1.29σ, respectively.

#### 2.4.2. Regional Warm Winter

Regions with more than 50% of the total number of stations estimated to have warm winter conditions are defined as regional warm winters. According to the proportion of the total number of effective stations, strong and weak warm winters can be divided.

### 2.5. Performance Metrics

This study uses four performance indicators (Table 2): correlation coefficient, root mean square error (RMSE), volume error (VE), and ratio of root mean square error and standard deviation (RSR) to evaluate the degree of deviation between model historical data and historical observation data [37,38]. Among them, the larger the correlation coefficient, the smaller the RMSE, VE, and RSR, the higher the consistency between the model and the observed data. The specific definition is as follows:

### 2.6. Trend Test

The Mann–Kendall (MK) method, which take the influences of self-correlation in extreme temperature into consideration comparing to other trend test methods, was utilised to estimate the trend and significance of the indices and temperature. The MK test does not require samples to follow a certain distribution, nor is it disturbed by a few outliers. In the MK test, the null hypothesis H0 is the time-series data and is the sample of *n* independent random variables with the same distribution; the alternative hypothesis H1 is a bilateral test, and for *k*, *j* ≤ *n*, and *k* ≠ *j*, the distributions of $x_{k}$ and $x_{j}$ are not the same. If the null hypothesis is unacceptable, there is an evident upward or downward trend in the time-series data at the α confidence level. For the statistics *Z*, a value greater than 0 indicates an upward trend, and a value less than 0 indicates a downward trend. The *Z*-score was calculated using the following formula:

(8) $$S=\overset{n-1}{\underset{k=1}{\sum }}\overset{n}{\underset{j-k+1}{\sum }}sgn\left(x_{j}-x_{k}\right)$$

(9) $$Var\left(s\right)=\frac{\left[n\left(n-1\right)\left(2n+5\right)-\sum_{j=1}^{m}t_{j\;}\left(t_{j}-1\right)\left(2t_{j}+5\right)\right]}{18}$$

(10) $$Z=\{\begin{array}{c} \frac{S-1}{\sqrt{Var\left(S\right)}}\;\;,\;\;\;S>0\\ 0\\ \frac{S+1}{\sqrt{Var\left(S\right)}}\;,\;S<0 \end{array}$$

## 3. Results

### 3.1. GCMs Evaluation and Prediction

**Figure 2:**
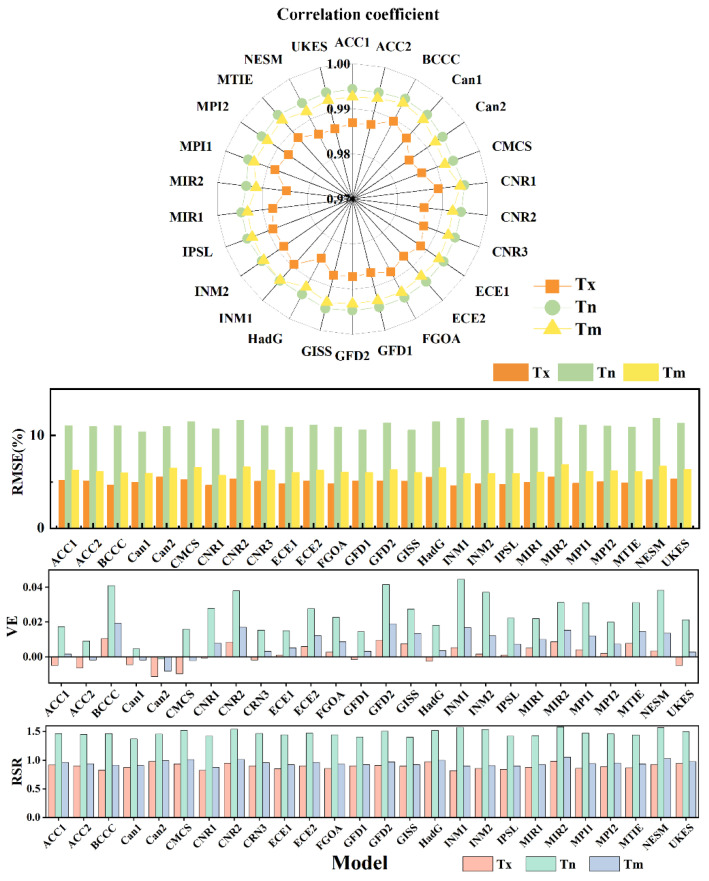
Performance metrics between CMIP6 model and observation data. maximum temperature (Tx), mean temperature (Tm), and minimum temperature (Tm).

All 26 site-scale climate models downscaled by the NWAI-WG method were verified according to the historical data of corresponding stations from 1961 to 2010; the results are shown in Figure 2. Pearson product-moment correlation showed that correlation coefficient and root mean square error (RMSE) ranged from 0.984 to 0.989 and 4.581 to 5.509 for maximum temperature and 0.993 to 0.995 and 10.352 to 11.906 for minimum temperature, respectively. The correlation coefficients of all models after scaling down were higher than 0.95, whereas RMSE showed a very high fitting relationship for maximum and minimum temperature attributes. The absolute value of VE index predicted by all models for temperature were less than 0.05. The RSR performance of all models is less than 1.6, with a mean of 1.1, and the RSR of the Tx is less than 1. This shows that the statistical downscaling method we used can restore the real data with high fidelity. The multi-model ensemble (MME) median CMIP6 data were obtained after downscaling the model.

**Figure 3:**
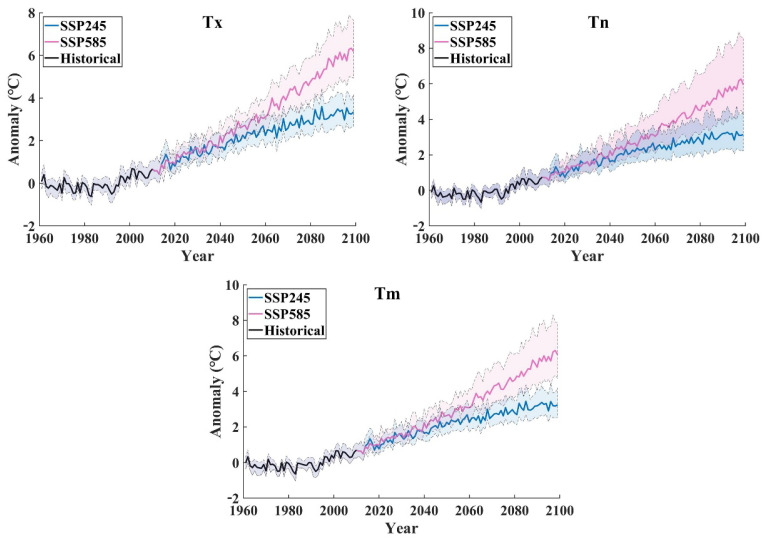
Time series of winter temperature anomaly changes. The fill range between dotted lines for all model, and the solid line represents the MME.

**Figure 4:**
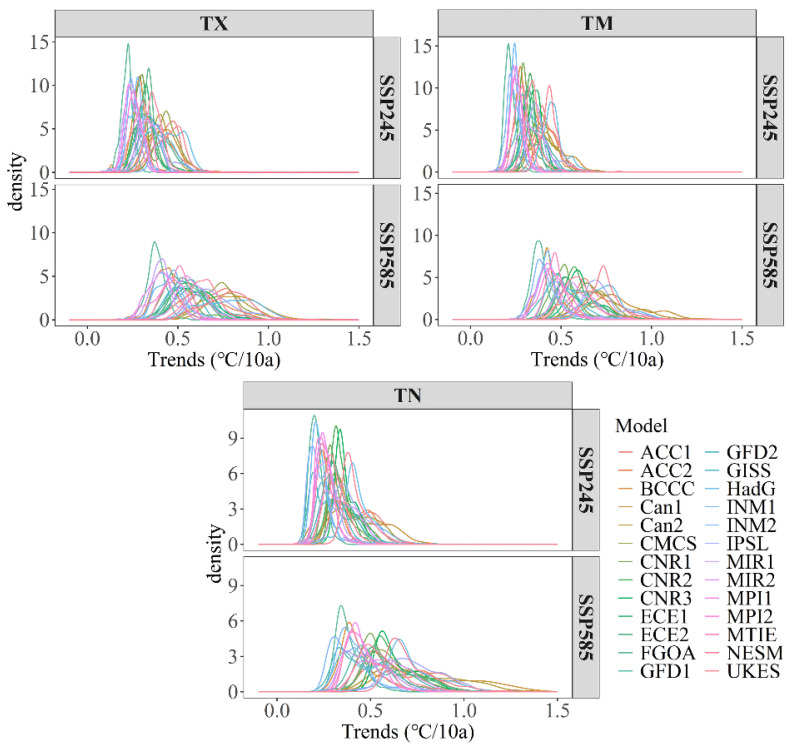
Probability density distribution of temperature growth rate at stations under different scenarios.

To estimate how winter temperature responds to global warming, we calculated the speed of temperature increase throughout the entire Chinese mainland and seven subregions. The results showed increasing trends of temperature indices, including maximum temperature, minimum temperature, and daily average temperature, as shown in all climate models under the Shared-Socioeconomic Pathway-Representative Concentration Pathway (SSP-RCP) scenario. Throughout the entire Chinese mainland, the historical record shows the increasing velocity of Tx and Tn of 0.02 °C/10a (Celsius per decade) and 0.09 °C/10a, respectively, during the period 1961–2010. The simulating forecast data shows that Tx and Tn warming velocities were 0.309 °C/10a and 0.298 °C/10a under the SSP245 scenario and 0.594 °C/10a and 0.492 °C/10a under the SSP585 scenario, respectively (Figure 3). Under the SSP245 scenario, all models show a small difference in the probability density distributions of site temperature growth rates. In the SSP585 scenario, the growth rate gap between sites increases (Figure 4). The maximum warming rate was slightly higher than the minimum temperature, but the regional difference in the minimum temperature growth rate was larger. For subregions, rapid temperature rise in winter was observed primarily in NC, NW, and NE, where the temperature is relatively low, whereas the temperature rise in SC is slower than that in other regions. Under the SSP245 scenario, the increasing rates of Tx, Tn and Tm were 0.309, 0.298 and 0.304, respectively. Under the SSP585 scenario, the increasing rates of Tx, Tn, and Tm were 0.594, 0.492 and 0.652, respectively (Table 3). To evaluate the degree of spatial heterogeneity, we calculated annual coefficient of variation of maximum temperature (Tx), mean temperature (Tm), and minimum temperature (Tn) converted to the Kelvin scale for the entire Chinese mainland.

**Figure 5:**
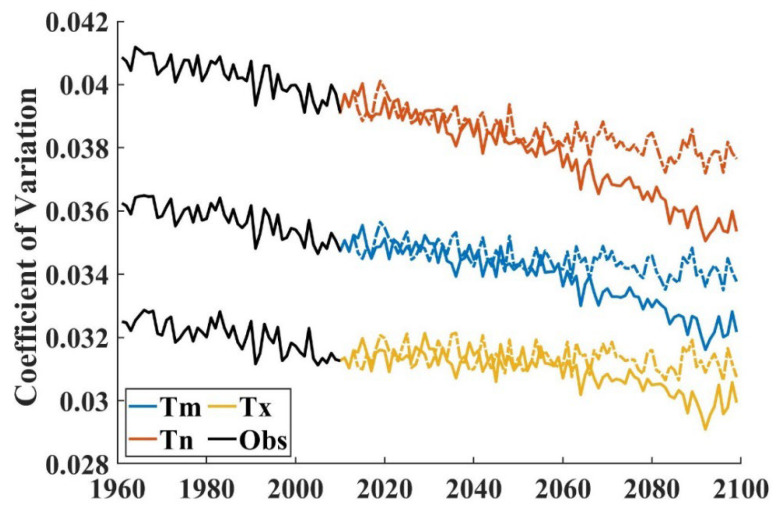
Temporal variation of MME coefficients of variation. The solid line represents the SSP585 scenario and the dashed line represents the SSP245 scenario.

As shown in Figure 5, the coefficients of variation decreased significantly during the period 1960–2100 (*p* < 0.05). However, the other indices decreased at different velocities. Minimum temperature increased rapidly compared to mean and maximum temperatures. The coefficient of variation for maximum temperature under the SSP585 scenario showed a downward trend of −1.71 × 10^−5^, which has higher radiation forcing and shows a faster decline compared with the downward trend of the coefficient of variation for maximum temperature of −0.89 × 10^−6^ under the SSP245 scenario. The coefficient of variation for minimum temperature under the SSP585 scenario showed a downward of −3.99 × 10^−5^ compared to a downward trend of −2.45 × 10^−5^ under the SSP245 scenario. The coefficient of variation for mean temperature also showed downward trends of −2.85 × 10^−5^ and −1.67 × 10^−5^ under the SSP585 and SSP245 scenarios, respectively. This shows that the temperature difference between the north and south is generally more likely to be smaller in the future.

### 3.2. Changes in Winter Extreme Climate Events

Both TXx and TNn showed upward trends, with the fastest increase observed for TXx. The inter-regional relative growth rate under the SSP585 scenario is consistent with that under the SSP245 scenario, and NE has the fastest growth rate for both indices. TX90P also showed an increasing trend, with the fastest growth rate observed in Northwest, North, and NE. In contrast, TN10P showed a decreasing trend, and the decline rate was fastest in the Southwest, South, and the QT Plateau regions of China. The DTR increasing trend was shown in the majority of the regions in China, but a downward trend was observed in the northeast part of Northwest IM. The variance data showed that the difference in the minimum temperature increasing trend may be the main reason for this phenomenon.

**Figure 6:**
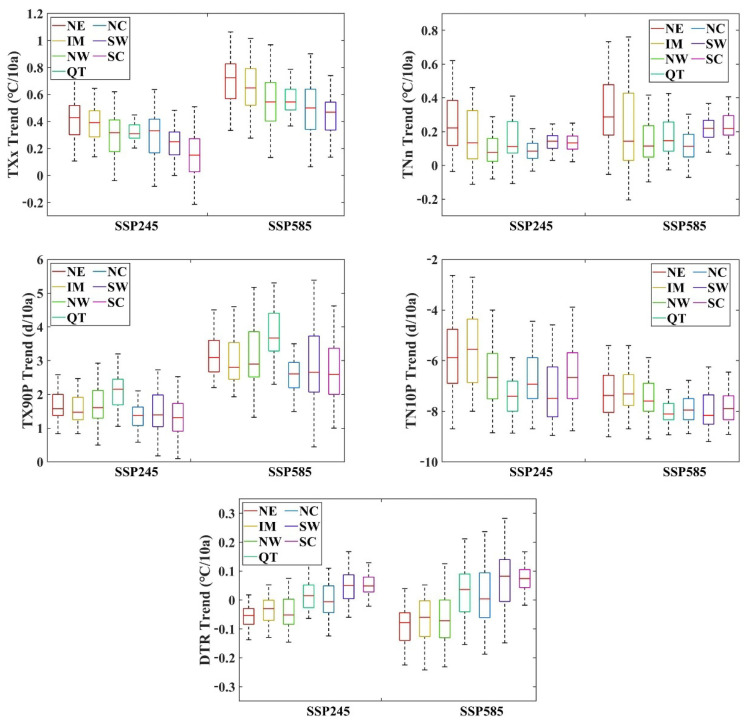
Temporal variation trend of extreme climate index. The whiskers in the box plots show the 10th and 90th percentiles and upper and lower boundaries show the 25th and 75th percentiles.

**Figure 7:**
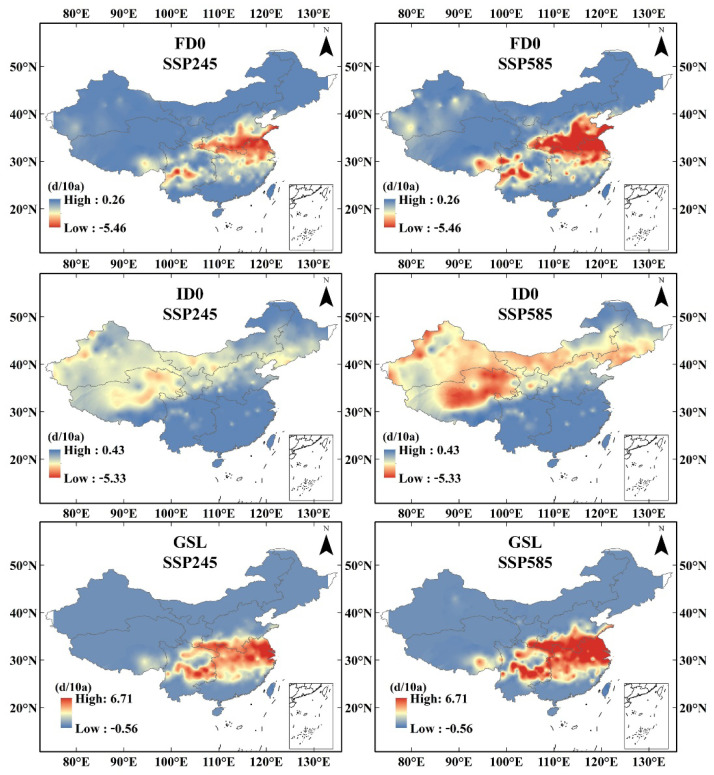
Spatial variation trend of extreme climate indices.

The increase in the growing season length ranged from 0 to 0.606 days/10a and 0 to 0.703 days/10a under the SSP245 and SSP585 scenarios, respectively. The main crop growing area is Eastern China, where rice, which is the main crop in China, is grown. Under the background of a stronger forcing pathway, the area facing the growing season length increase expanded northwards. This indicates that global warming has provided sufficient temperature for winter crop growth in Eastern China. FD0 decreased primarily in Eastern and Southwestern China but increased slightly or remained unchanged in other parts of China. The trend of FD0 ranged from −0.453 to 0 days/10a and −0.563 to 0 days/10a under the SSP245 and SSP585 scenarios, respectively. The significantly reduced area showed an enlargement and a northward shift under the SSP585 scenario. The decrease in ice days (ID0) was primarily concentrated in NW and IM, where the temperature was already low. The decreasing trend of ID0 ranged from −0.364 to 0 days/10a and −0.535 to 0 days/10a under the SSP245 and SSP585 scenarios, respectively. Under the SSP585 scenario, the ID0 index in NE also showed a significant and rapid decline. Changes in these two indices were primarily observed in places where the original temperature is relatively low and reflect the changes in the lowest and highest temperatures, respectively (Figure 6 and Figure 7).

**Figure 8:**
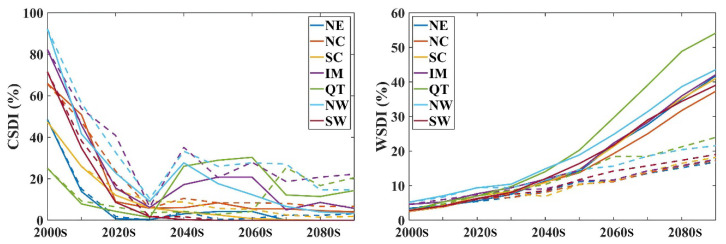
The CSDIs and WSDIs for each decade in the 21st century. The solid line represents the SSP585 scenario, and the dashed line represents the SSP245 scenario.

The dynamic baseline method was utilised to calculate the warm spell duration time (WSDI) and cold spell duration time (CSDI) taking into consideration the warming background while defining the threshold. These two indices show different responses to global warming. For the entire Chinese mainland, before the mid-21st century, the WSDI showed a synchronous increasing trend under both warming scenarios. However, after the 2050s, the WSDI under the SSP245 scenario maintained a consistent velocity until the end of this century, whereas the WSDI under the SSP585 scenario accelerated from the 2050s to the 2090s. The CSDI showed a similar trend in the 21st century. In all warming scenarios, the CSDI ratio decreases to less than 20% per year and maintains a low standard. Among the regions, the QT Plateau showed the largest discrepancy in the WSDI between the SSP245 and SSP585 scenarios. These results show that the QT Plateau is more sensitive to warming. The trend in the CSDI shows different bias between the SSP245 and SSP585 scenarios, with a deceasing trend observed in two radiation forcing pathways. The CSDI frequency in each region decreased to less than 10% until the 2030s, with the fastest decrease observed in NW compared to that seen in other regions. However, after the 2030s, the CSDI increased slightly in some regions (Figure 8).

Our prediction revealed that the warm-relevant index presented a stronger response to higher radiation forcing. There was a slight difference in the cold correlation index under different radiation forcing scenarios. This indicates that the change in the highest temperature in winter was more sensitive to different radiation forces. The effectiveness of a higher radiation force will lead to a tremendous increase in the WSDI after the 2050s compared with that of the SSP245 scenario.

### 3.3. Warm Winter Event

**Figure 9:**
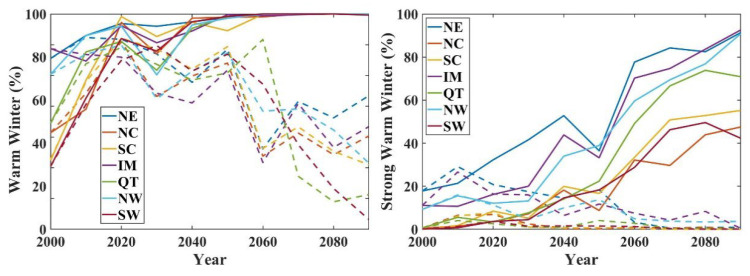
Temporal variations of warm winter events ratio in each region. The solid line represents the SSP585 scenario, and the dashed line represents the SSP245 scenario.

**Figure 10:**
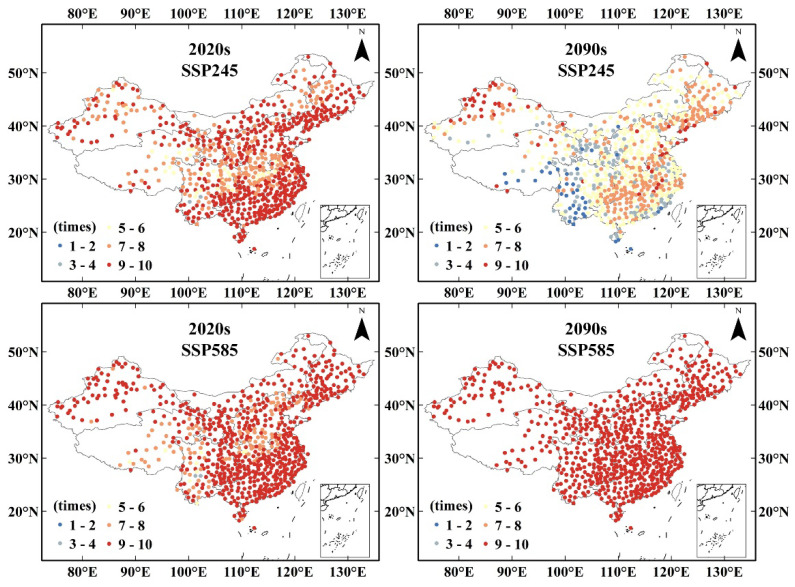
Spatial variation of warm winter events frequency predictions in the 2020s and 2090s under the SSP245 and SSP585 scenarios.

The warm winter index announced by the CMA was applied to estimate the warm bias during the entire season, which has seldom been considered in previous studies. Warm winter can be divided into two classes, i.e., warm winter and strong warm winter, according to its intensity and whether the average temperature exceeds one or two standard deviations more than the climatological normal calculated from the time series. The regional warm winters represent the majority bias in the subregions. The statistics of warm winter events per decade showed a different variation path between the SSP245 and SSP585 scenarios. In the period 2000–2030, all regions show a rapidly increasing trend of warm winter frequency under both SSP245 and SSP585 scenarios, especially in NE, NW, and IM, which experience high frequencies of warm winters at a rate of more than eight times per decade. However, after the 2030s, a steady high frequency of warm winters was detected only under the SSP585 scenario, whereas the warm winter frequency decreased in all regions under the SSP245 scenario (Figure 9). The frequency of strong warm winters showed an increasing trend in the 21st century by approximately 7 times/10a in the regions of NE, NW, QT Plateau, and IM under the SSP585 scenario but remains at a low level under the SSP245 scenario (Figure 10). This reveals an avoided approach from a strong, warm winter event. This could be due to the change in the climatic reference value, which matches the change in temperature, leading to a relatively moderate temperature rise in the later period. Under the SSP585 scenario, the temperature increased even more rapidly.

### 3.4. Population Exposure to Warm Winter Events

**Figure 11:**
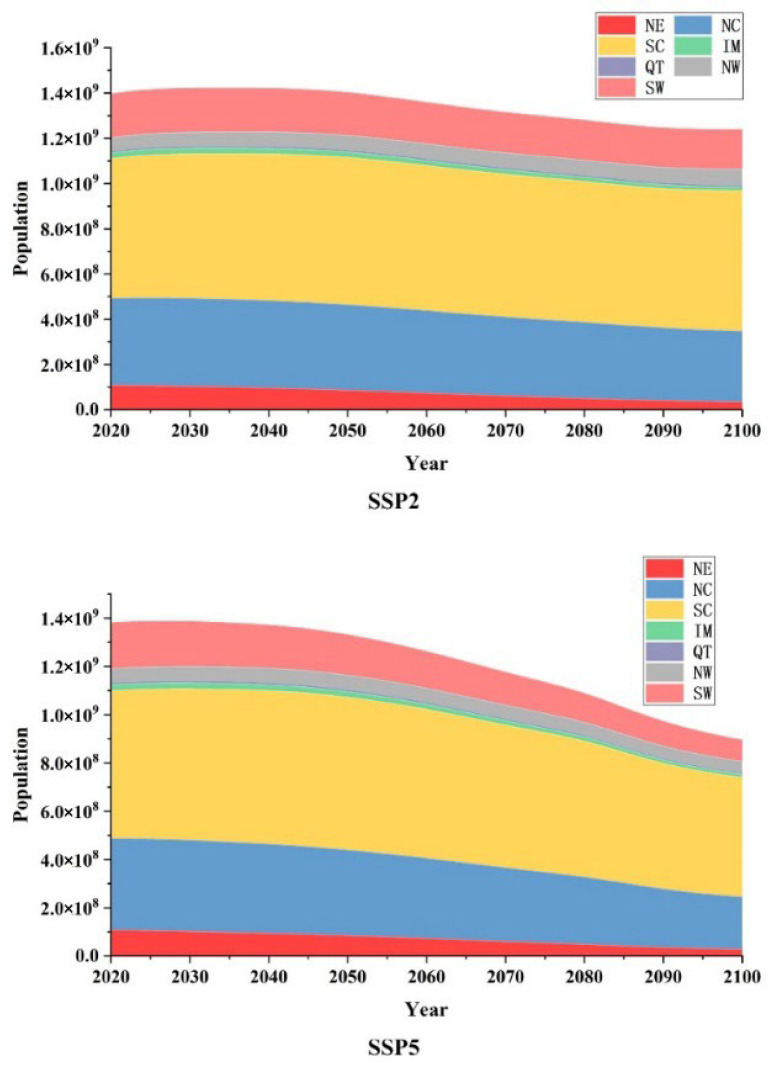
Population in each region under future scenarios.

The shared socioeconomic pathways (SSP) data was utilised to estimate population exposure to warm winter events on the Chinese mainland under future scenarios [39]. From a national perspective, under the two scenarios of SSP2 and SSP5, the overall population of the Chinese mainland showed a downward trend, of which the overall national population change trends were −26.318 million/10a and −61.419 million/10a, respectively. SSP population data showed that Chinese population is mainly distributed in NC, SC and SW. In 2010, the proportion of the population in these regions was 27.4%, 44.5% and 14.2%, respectively, while the population in QT region accounted for only 0.65%. According to the estimates of the two scenarios, by 2100, the population of SC will exceed half of the country’s, reaching 50.07% in the SSP2 scenario and 55.23% in the SSP5 scenario. Different population changes were shown under the two scenarios (Figure 11). Under the SSP2 scenario, except for the NW, which showed a growth trend of 124,500 people/10a, the remaining six sub-regions all showed a downward trend. Among them, the decline rates in the NE and NC regions were more prominent, which were −1.021 and −1.049 million people/10a, respectively. In the SSP5 scenario, all sub-regions showed a significant decreasing trend, among which the NE, NC, SC and SW regions all showed higher population reduction rates, −1.054, −2.04, −1.354, and −1.349 million people/10a, respectively. Table 4 compares the increase in the rate of population change in the SSP5 scenario compared to the SSP2 scenario. The results show that the population change trends in SC, QT, NW, and SW are quite different under the two SSP scenarios at 501.33%, 624.68%, −199.74%, 378.20%, respectively. It shows that these regions are more sensitive to the challenges of climate change mitigation and adaptation and are important research areas that affect future climate forcing.

**Figure 12:**
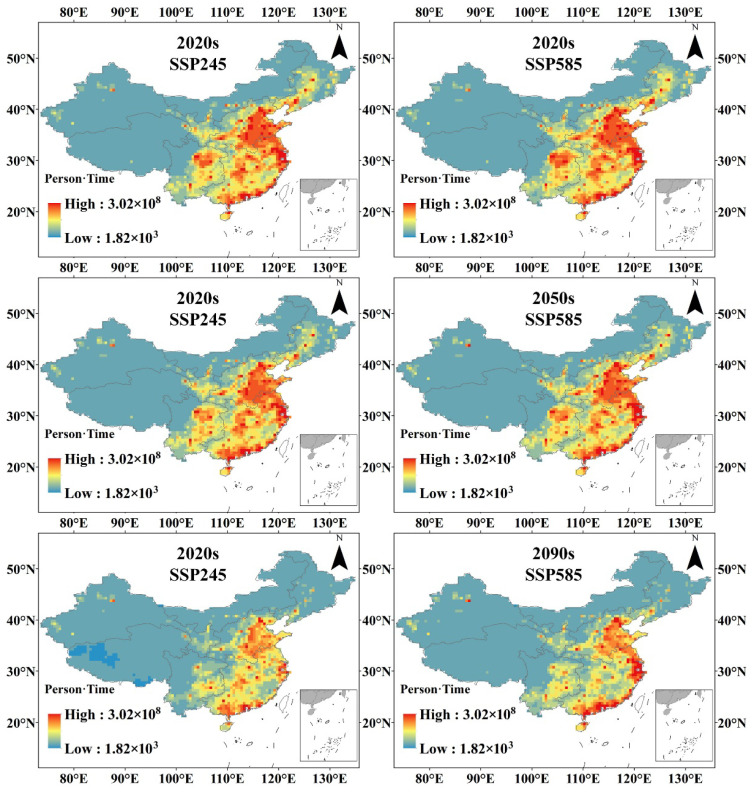
Spatial distribution of population exposure to warm winter events.

Based on the population data under the SSP2 and SSP5 scenarios and the frequency of future warm winter events under the SSP245 and SSP585 scenarios, the population exposure to the warm winter events on the Chinese mainland under the two scenarios was calculated, and a decade-by-decade study on the population exposure to the warm winter events was conducted in each sub-region population exposure statistics. Figure 12 reflects the spatial distribution of population exposure to the national warm winter event at the beginning of this century (2020s), the middle of this century (2050s), and the end of this century (2090s). The results show that the number of people exposed to warm winters in China is mainly distributed in the eastern part of the Hu-Huanyong line, especially in NC, SC and SW. Beijing-Tianjin-Hebei, Yangtze River Delta, Pearl River Delta, urban agglomeration in the middle reaches of the Yangtze River, and Chengdu-Chongqing urban agglomeration are the areas where the number of people exposed to the warm winter event is the most concentrated due to their large populations. At the beginning of this century, in the forecast results of SSP2 and SSP5, the population exposure to warm winter events on the Chinese mainland was 12.634 billion person-times and 13.354 billion person-times, respectively. The population exposure to SC and NC accounted for a larger proportion, reaching 6.122 and 3.665 billion, accounting for 45.84% and 27.44%, respectively. In the middle of the century, the overall population exposure declined to 11.999 billion and 12.726 billion in SSP2 and SSP5 scenarios, respectively. Among the sub-regions, the proportion of South China increased to 48.07% and 46.57%, respectively, and the population exposure and proportion of other regions showed a downward trend. By the end of this century (2090s), the population exposure to warm winter events under the SSP245 scenario will be greatly reduced, reaching 6.937 billion person-times, which is about 54.9% of the 2020s. To 242 million in the 2090s, the decline was about 74.82%, and the NW had the least reduction, from 517 million in the 2020s to 368 million in the 2090s, a decrease of about 28.95%. Under the SSP585 scenario, the population exposure to the national warm winter event in the 2090s was 9.358 billion person-times, a decrease of about 29.97%, and the Northeast region decreased the most, from 1.018 billion person-times to 314 million person-times, a decrease of about 69.16%, and other regions decreased less than 50% (Table 5 and Table 6).

## 4. Discussion

**Figure 13:**
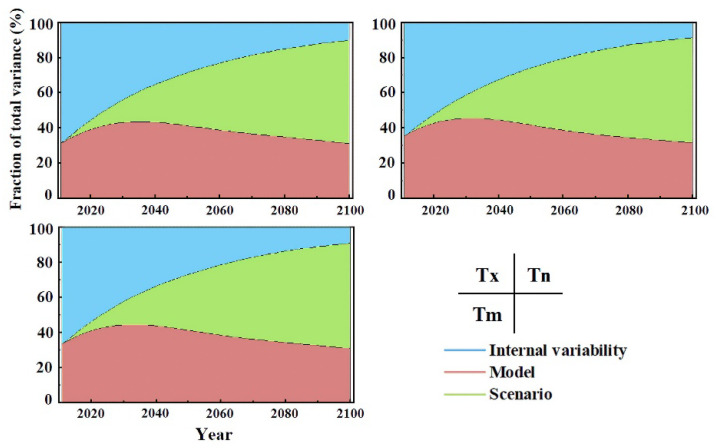
Changes in the proportion of the three uncertainties.

In summary, the accuracies of 26 GCMs were evaluated. The result showed that the GCM historical data have high goodness of fit with the observation data, with the Pearson product-moment correlation coefficient and RMSE ranging from 0.984 to 0.989 and 4.581 to 5.509 for maximum temperature and 0.993 to 0.995 and 10.352 to 11.906 for minimum temperature, respectively. The uncertainty of CMIP6 data is due to three factors: internal variation, model uncertainty, and scenario uncertainty. Internal uncertainties of maximum and minimum temperatures showed a consistent decreasing trend from approximately 70% in 2010 to 10% in 2100. Scenario uncertainty, which is the dominant uncertainty after the 2060s, showed a rapidly increasing trend during the study period. Model uncertainty decreased slightly but was observed to be greatest until the 2060s (Figure 13).

**Figure 14:**
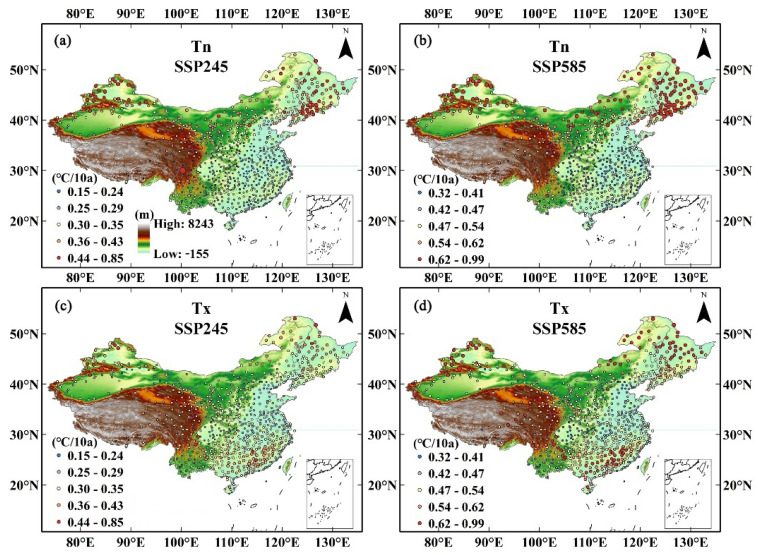
Rates of increase in maximum and minimum temperatures under two SSP scenarios. (**a**) Tmin, SSP245; (**b**) Tmin, SSP585; (**c**) Tmax, SSP245; (**d**) Tmax, SSP585.

It was found that maximum and minimum temperatures increased at different rates under a warming scenario. Average minimum temperature of the entire Chinese mainland increased by 0.298 °C/10a slower than the maximum temperature under the SSP245 scenario compared to 0.493 °C/10a under the SSP585 scenario. According to this study, NE, IM, and NW showed higher minimum temperature increasing rates, but the maximum temperature increased faster in SC and NE than in other subregions (Figure 14). Furthermore, the variation coefficient reveals that the temperature difference between the south and north is smaller. We calculated the relationship between the warming rates for maximum and minimum temperatures and the altitudes of the observation stations under different conditions. As shown in Table 7, there was a significant, positive correlation between the increase rate of maximum temperature and elevation in SW, a certain correlation in NC and SC, a negative correlation in IM, and a weak correlation in other regions. The increasing rate of minimum temperature is positively correlated with elevation in NE and negatively correlated with elevation in NW, which may be due to the increase in short-wave radiation sources in NW because of the distribution of deserts in basins in this region [40]. Due to the lack of high-altitude meteorological stations over the QT Plateau, elevation-dependent warming cannot be observed.

Frequent and intense ETIs show a significant response to future warming. The intensity and frequency of the extreme cold index decreased more in NW, NC, and the QT Plateau. The extreme hot index in winter increased significantly under both warming scenarios. TN10p decreased by −0.651 and −0.761 days, TNn increased by 0.149 °C and 0.215 °C, TX90p increased by 0.153 and 0.297 days, and TXx increased by 0.259 °C and 0.489 °C under the SSP245 and SSP585 warming scenarios, respectively. A rapid and consistent decreasing trend of CSDI is shown in all subregions on the Chinese mainland under both SSP245 and SSP585 scenarios. The WSDI shows a consistently increasing trend under the SSP245 scenario but shows saltation acceleration after the 2050s under the SSP585 scenario. Maximum temperature is more sensitive to global warming, and the reduction in radiative forcing helps mitigate the warming trend of maximum temperature.

Winter average temperature bias was estimated using the warm winter index announced by the CMA. During the period 2000–2030, warm winter frequency shows a rapid increase under both SSP245 and SSP585 scenarios, but a decrease in warm winter frequency is shown after the 2030s under the SSP245 scenarios. Cold winter frequency decreases very rapidly and will remain at a low level in the future. These results reveal that warm winter events can be prevented under the SSP245 pathway. SW, QT, and IM are areas less affected by warm winters under the SSP245 scenario in the future. Warmer winters are more frequent in the eastern seaboard and arid north-western regions, which may be primarily attributed to the response of the East Asian winter monsoon to global warming [41,42]. Population exposure shows a downward trend in both scenarios. While the population declined faster in the ssp585 scenario, the population exposure declined more in the SSP245 scenario. This is mainly because in the SSP585 scenario, although the population shows a decreasing trend, the overall warm winter frequency and impact range are wider, while in the SSP245 scenario, the population number decreases slightly, but the overall number and scope of warm winters are significantly weakened, so the population exposure to warm winter events has a relatively large reduction. Therefore, we should strictly control carbon emissions and promote green production and lifestyle, so that the greenhouse gas emission path is controlled in the SSP245 scenario, which can avoid 25.87% of the population exposure risk in warm winters.

Extreme winter temperatures have many negative and positive impacts on agricultural production. SC is the main rice-growing regions in China. The majority of regions with maximum temperature increase are located in these regions, with the warming trend anticipated to lead to longer growing seasons and more accumulated temperature in the future. However, NE is the main wheat and rice farming region, which shows a significant increase in minimum temperature that will have a negative influence on spring phenology in future [43,44,45]. In future studies, we will pay more attention to the influence of winter atmospheric circulation on warming as well as the influence of winter warming on crop production and human health.

## 5. Conclusions

In this study, we used 26 GCM datasets from the entire Chinese mainland derived from the CMIP6 datasets, which include maximum and minimum temperatures during the period of 1961–2100 under the SSP245 and SSP585 warming scenarios. The NWAI-WG method, which is a statistical downscaling method combining observation data, was utilised to downscale the gridded data to the station scale to enhance precision. Nine ETIs were estimated from 2010 to 2100 based on the multi-model ensemble-predicted data after checking for robustness and uncertainty. The temporal and spatial variations were calculated using the MK method and the variable coefficient, respectively. The frequency of future warm winter events was calculated by the warm winter index proposed by CMA. The population exposure to warm winter events was calculated by combining the SSP population forecast data.

Under the background of global warming, the national winter temperature shows a significant growth trend. Compared with other seasons and the whole, winter also showed asymmetric temperature rise, but the heating rate of the maximum temperature in winter was slightly higher than that of the minimum temperature. The coefficients of variation calculated based on the maximum temperature, minimum temperature, and average temperature all showed a significant decreasing trend, indicating that the region with low temperature had a faster heating rate and the north–south temperature difference decreased.

The extreme climate indices calculated according to the ETCCDI indicator showed different trends, with a significant decrease in cold-related extreme climate events and a significant increase in warm-related extreme climate events. The warm winter conditions showed a large difference between the SSP245 and SSP585 conditions. Under the SSP245 scenario, by the end of this century, the frequency of warm winters will decrease and remain at a low level. The Southwest, Qinghai-Tibet and Inner Mongolia regions will decrease from 7–9 times/10a in the early 21st century to less than 4 times/10a, while in the SSP585 scenario, the frequency of warm winter events will reach the highest, reaching 8–10 times/10a.

The overall population of the Chinese mainland shows a downward trend. The overall population change trends in the country are −26.32 million and −61.42 million/10a, respectively. The population decrease trend in NE, NC, SC, and SW is relatively high. In the future scenario, the population exposure to warm winters on the Chinese mainland shows a decline. By the 2090s, the population exposure to warm winter events in the SSP245 scenario will be 6.937 billion person-times, while that in the SSP585 scenario will be 9.358 billion person-times. The SSP245 scenario has a much lower exposure risk than the SSP585 scenario due to the decreasing frequency of both population and warm winter events.

The intensity and frequency of future extreme weather events show an increasing trend under the two warming scenarios. Warm winter events decreased under SSP245 and increased under SSP585. Population exposure showed a downward trend. A consistent warming trend was observed on the Chinese mainland under all SSPs in the 21st century, and stricter emission reduction policies can help reduce the negative impact of global warming on winter temperatures.

## Figures and Tables

**Table 1:** Selected extreme temperature indices.

Label	Index Name	Index Definition	Units
FD0	Frost days	Number of days with minimum temperature < 0°	days
ID0	Ice days	Number of days with maximum temperature < 0°	days
TN10p	Cold night	The number of days on which the daily minimum temperature was less than the 10th percentile in the last 30 years	days
TX90p	Warm day	The number of days on which the daily maximum temperature was greater than the 90th percentile in the last 30 years	days
TNn	Minimum value of daily minimum temperature	Minimum value of daily minimum temperature	°C
TXx	Maximum value of daily maximum temperature	Maximum value of daily maximum temperature	°C
CSDI	Cold spell duration index	Count at least six consecutive days with TN below the 10th percentile	days
WSDI	Warm spell duration index	Count at least six consecutive days with TX over the 90th percentile	days
DTR	Daily temperature range	The difference between maximum temperature and minimum temperature	°C
GSL	Growth season length	Span of at least 6 days with daily mean temperature over 5 °C	days

**Table 2:** Performance metrics definition. *Obs* is the observed value, M is modelled.

Performance Metrics	Definition	Range
Correlation coefficients	$r=\frac{N\sum Obs_{i}M_{i}-\sum Obs_{i}\sum M_{i}}{\sqrt{N\sum Obs_{i}{}^{2}-{\left(\sum Obs_{i}\right)}^{2}}\sqrt{N\sum M_{i}{}^{2}-{\left(\sum M_{i}\right)}^{2}}}$	[−1, +1]
Root mean square error	$\mathrm{RMSE}=\sqrt{\frac{1}{N}\overset{N}{\underset{i=1}{\sum }}{\left(Obs_{i}-M_{i}\right)}^{2}}$	[0, +∞]
Volume error	$\mathrm{VE}=\frac{\sum_{i=1}^{N}Obs_{i}-\sum_{i=1}^{N}M_{i}}{\sum_{i=1}^{N}Obs_{i}}$	[−∞, +∞]
Ratio of root mean square error and standard deviation	$\mathrm{RSR}=\frac{\sqrt{\frac{1}{N}\sum_{i=1}^{N}{\left(Obs_{i}-M_{i}\right)}^{2}}}{\sqrt{\frac{1}{N}\sum_{i=1}^{N}{\left(Obs_{i}-\bar{Obs}\right)}^{2}}}$	[0, +∞]

**Table 3:** Temperature increasing trend in each region.

	Scenario	NE	NC	SC	IM	QT	NW	SW	ECM
Tx	SSP245	0.345	0.280	0.313	0.322	0.301	0.317	0.292	0.309
	SSP585	0.581	0.452	0.507	0.541	0.503	0.525	0.474	0.594
Tn	SSP245	0.409	0.276	0.254	0.363	0.290	0.366	0.249	0.298
	SSP585	0.682	0.443	0.423	0.615	0.486	0.595	0.405	0.492
Tm	SSP245	0.377	0.279	0.284	0.342	0.296	0.338	0.271	0.304
	SSP585	0.632	0.449	0.467	0.578	0.495	0.56	0.441	0.652

**Table 4:** Population trend of each region from 2020 to 2100 (10,000 people/10 years).

	SSP2	SSP5	Ratio
NE	−1021.5	−1054.6	3.24%
NC	−1049.8	−2040.7	94.39%
SC	−225.3	−1354.6	501.24%
IM	−126.5	−158.6	25.38%
QT	−4.0	−28.7	617.50%
NW	124.5	−124.2	−199.76%
SW	−282.3	−1349.8	378.14%
ECM	−2631.8	−6141.9	133.37%

**Table 5:** Population exposure to warm winter events under the SSP2 scenario (100 million person-times).

	2020s	2030s	2040s	2050s	2060s	2070s	2080s	2090s
NE	9.63	8.73	7.12	7.13	4.42	3.53	3.38	2.42
NC	35.21	28.57	30.37	31.17	21.56	18.92	18.09	19.33
SC	57.90	56.95	53.59	57.68	37.28	38.28	34.39	35.97
IM	2.16	1.87	1.75	1.86	1.28	1.23	1.13	0.98
QT	0.84	0.82	0.78	0.80	0.90	0.49	0.36	0.45
NW	5.17	4.46	5.17	5.59	4.53	4.64	4.04	3.68
SW	16.53	17.44	15.69	16.15	14.02	10.42	7.57	7.27
ECM	126.34	118.15	113.75	119.99	88.68	79.05	70.59	69.37

**Table 6:** Population exposure to warm winter events under the SSP5 scenario (100 million person-times).

	2020s	2030s	2040s	2050s	2060s	2070s	2080s	2090s
NE	10.18	9.42	8.75	7.85	6.69	5.45	4.21	3.14
NC	36.65	33.20	35.24	34.11	31.71	29.34	26.22	22.92
SC	61.22	58.63	61.77	59.27	60.49	57.99	54.42	50.78
IM	2.41	2.30	2.19	2.23	2.01	1.80	1.58	1.38
QT	0.84	0.77	0.90	0.91	0.88	0.83	0.77	0.71
NW	5.58	4.82	5.65	5.77	5.66	5.43	5.13	4.85
SW	17.38	16.12	16.92	15.95	14.50	12.90	11.21	9.58
ECM	133.54	124.03	131.02	127.26	122.30	113.99	103.77	93.58

**Table 7:** Correlation coefficient between elevation and rate of increase in temperature.

		NE	NC	NS	IM	QT	NW	SW
Tmax	SSP245	0.197	0.436	0.339	−0.374	0.072	0.120	0.482
	SSP585	0.236	0.389	0.336	−0.327	0.150	0.002	0.559
Tmin	SSP245	0.328	0.041	0.015	0.096	−0.168	−0.542	0.121
	SSP585	0.462	0.026	0.046	0.164	−0.090	−0.571	0.099

## Data Availability

The daily meteorological data was provided by the National Meteorological Information Center (NMIC), China Meteorological Administration (CMA) (http://data.cma.cn/ accessed on 5 November 2019). The CMIP6 data were collected from the CMIP6 website (https://pcmdi.llnl.gov/CMIP6/ accessed on 9 September 2020) and De Li Liu of the NSW Department of Primary Industries used NWAI-WG to downscale downscaled daily data. The population grid point data (2010–2100) of Chinese mainland under the Shared Socioeconomic Pathways (SSPs) used in this paper are from the team of Professor Jiang Tong, Institute of Disaster Risk Management, Nanjing University of Information Science and Technology.
